# Microvascular proliferation is associated with high tumour blood flow by mpMRI and disease progression in primary prostate cancer

**DOI:** 10.1038/s41598-023-45158-4

**Published:** 2023-10-20

**Authors:** Astrid Børretzen, Lars A. R. Reisæter, Anders Ringheim, Karsten Gravdal, Svein A. Haukaas, Kristine E. Fasmer, Ingfrid H. S. Haldorsen, Christian Beisland, Lars A. Akslen, Ole J. Halvorsen

**Affiliations:** 1 Centre for Cancer Biomarkers CCBIO, Gade Laboratory for Pathology, Department of Clinical Medicine, University of Bergen, Bergen, Norway; 2 Department of Pathology, Haukeland University Hospital, 5021 Bergen, Norway; 3 Department of Clinical Medicine, University of Bergen, Bergen, Norway; 4 Department of Radiology, Haukeland University Hospital, Bergen, Norway; 5 Department of Urology, Haukeland University Hospital, Bergen, Norway; 6 Mohn Medical Imaging and Visualization Centre (MMIV), Department of Radiology, Haukeland University Hospital, Bergen, Norway

**Keywords:** Prostate cancer, Prognostic markers

## Abstract

Active angiogenesis may be assessed by immunohistochemistry using Nestin, a marker of newly formed vessels, combined with Ki67 for proliferating cells. Here, we studied microvascular proliferation by Nestin-Ki67 co-expression in prostate cancer, focusing on relations to quantitative imaging parameters from anatomically matched areas obtained by preoperative mpMRI, clinico-pathological features and prognosis. Tumour slides from 67 patients (radical prostatectomies) were stained for Nestin-Ki67. Proliferative microvessel density (pMVD) and presence of glomeruloid microvascular proliferation (GMP) were recorded. From mpMRI, forward volume transfer constant (K^trans^), reverse volume transfer constant (k_ep_), volume of EES (v_e_), blood flow, and apparent diffusion coefficient (ADC) were obtained. High pMVD was associated with high blood flow (*p* = 0.008) and low ADC (*p* = 0.032). High K^trans^, k_ep_, and blood flow were associated with high Gleason score. High pMVD, GMP, and low ADC were associated with most adverse clinico-pathological factors. Regarding prognosis, high pMVD, K^trans^, k_ep_, and low ADC were associated with reduced biochemical recurrence-free- and metastasis-free survival (*p* ≤ 0.044) and high blood flow with reduced time to biochemical- and clinical recurrence (*p* < 0.026). In multivariate analyses however, microvascular proliferation was a stronger predictor compared with blood flow. Indirect, dynamic markers of angiogenesis from mpMRI and direct, static markers of angiogenesis from immunohistochemistry may aid in the stratification and therapy planning of prostate cancer patients.

## Introduction

Angiogenesis, the sprouting of new vessels from existing blood vasculature is essential for tumour growth and metastasis^1,2^. Tumours may thus be treated by angiogenesis inhibitors, as hypothesized by Judah Folkman in 1971^3^. Histological biomarkers reflecting angiogenesis, such as microvascular density (MVD), could be useful prognostic indicators, as evaluated in numerous studies^4–7^. In prostate cancer, the results of these studies are somewhat conflicting^4,5,8–13^. MVD reflects inter-capillary distance and is determined by stimulating and inhibiting angiogenic factors as well as by the metabolic need of the tumour cells^4^. MVD is thus not a genuine marker specific for active angiogenesis nor the angiogenic dependence of the tumour tissue^4^. Measurement of vessels with proliferating endothelial cells by dual immunohistochemistry (IHC) with Factor VIII and Ki67 may be a better marker for ongoing tumour angiogenesis and outcome prediction^14–16^. Nestin is an intermediate filament protein found in neuroepithelial stem cells and glioma cells, but also in rapidly growing endothelial cells during active angiogenesis^17^. Hence, Nestin could be used as a marker of immature, newly formed vessels. Our group has used dual IHC by Nestin and Ki67 to assess proliferating immature blood vessels and its prognostic value in prostate cancer in a different cohort^18^, breast cancer^19^, and lung cancer^20^.

In prostate cancer, multiparametric MRI (mpMRI) including diffusion-weighted imaging (DWI) and dynamic contrast-enhanced imaging (DCE-MRI) add functional information to conventional T1-and T2-weighted anatomic imaging^21^. By measuring the uptake and washout of contrast on DCE-MRI, tumour angiogenesis can be estimated indirectly by mathematical pharmacokinetic models^22,23^. The rate of enhancement and washout of contrast is depending on the perfusion, the vascular permeability, and the volume of extravascular, extracellular space (EES) per unit volume of tissue (v_e_)^22^. Quantitative parameters such as the forward (K^trans^) and reverse (k_ep_) volume transfer constant between blood plasma and EES^24^) yield combined information on vascular permeability and perfusion and may hence reflect the tumour angiogenesis in vivo^22^.

Correlations between quantitative DCE-MRI-parameters and direct tissue-based biomarkers of angiogenesis, such as vascular endothelial growth factor (VEGF) and MVD have been studied in several malignancies, including in prostate cancer, with varying results^22,25–32^. The relationship between mpMRI-parameters and ongoing angiogenesis, estimated by microvascular proliferation, has been studied to a much lesser extent in clinical tumour samples^33^ and is to our knowledge not previously examined in prostate cancer.

Here, we evaluated microvascular proliferation by Nestin-Ki67 co-expression in radical prostatectomy specimens from 67 patients as a direct, static measure of ongoing angiogenesis. From anatomically matched areas obtained by preoperative mpMRI, the quantitative imaging parameters K^trans^, k_ep_, v_e_, and blood flow were studied as indirect, dynamic estimates of angiogenesis. In this study, we focused particularly on relations between microvascular proliferation and quantitative parameters from mpMRI and on potential associations with clinico-pathological features and patient outcome.

## Results

Descriptive statistics for the immunohistochemical markers of angiogenesis (microvessel density (MVD)/mm^2^, proliferative microvessel density (pMVD)/mm^2^ and vascular proliferation index (VPI = pMVD/MVD%) and parameters from mpMRI (forward volume transfer constant (K^trans^ [min^-1^]), reverse volume transfer constant (k_ep_ [min^-1^]), volume of EES (v_e_), blood flow (mL/100 g/min), and apparent diffusion coefficient (ADC [mm^2^/s])) are presented in Table 1. For all analyses, median was used as cut-off for all variables except blood flow (cut-off by lower quartile).

**Table 1:** Descriptive statistics for immunohistochemical markers of angiogenesis and quantitative mpMRI-parameters.

Variables	No. of patients	Mean	Median	Minimum	Maximum
MVD^1^	67	181.01	165.74	24.78	493.55
pMVD^2^	67	1.24	0.41	0.00	33.72
VPI^3^	67	0.66	0.22	0.00	15.40
K^trans4^	60	0.20	0.13	< 0.01	1.33
k_ep_^5^	60	0.29	0.17	0.04	2.00
v_e_^6^	60	0.72	0.90	< 0.01	1.00
Blood flow^7^	52	9.65	8.58	2.79	30.36
ADC^8^	67	807.43	807.00	369.00	1712.00

^1^Microvessel density/mm^2^ in pathological high-grade area.

^2^Proliferating microvessel density/mm^2^ in pathological high-grade area.

^3^Vascular proliferation index (%) in pathological high-grade area.

^4^ Median value of K^trans^ (min^-1^) in pathological high-grade area.

^5^ Median value of k_ep_ (min^-1^) in pathological high-grade area.

^6^ Median value of v_e_ in pathological high-grade area.

^7^ Mean value of blood flow (mL/100 g/min) in pathological high-grade area.

^8^Apparent diffusion coefficient (mm^2^/s) in pathological high-grade area.

### Tissue-based microvascular proliferation is associated with high tumour blood flow

High pMVD was associated with high tumour blood flow (*p* = 0.008), low ADC (*p* = 0.032) and with a trend for high k_ep_ (*p* = 0.063). High VPI was associated with low ADC and low v_e_ (both *p* = 0.038), with a trend for high tumour blood flow (0 = 0.054). MVD and GMP were not associated with any of the quantitative mpMRI-parameters (Table 2). High K^trans^ and high k_ep_ were associated with high blood flow (*p* = 0.001 and *p* = 0.010). Neither K^trans^, k_ep_ nor blood flow were associated with ADC.

**Table 2:** Associations between immunohistochemical markers of angiogenesis and quantitative mpMRI-parameters.

Variables	MVD^1^			pMVD^1^			VPI^1^			GMP^2^		
	Low	High	*p*-value^3^	Low	High	*p*-value^3^	Low	High	*p*-value^3^	Absent	Present	*p*-value^3^
	n (%)	n( %)		n (%)	n (%)		n (%)	n (%)		n (%)	n (%)	
K^trans4^			0.3			0.43			1			0.47
Low	17 (57)	13 (43)		13 (43)	17 (57)		14 (47)	16 (53)		27 (90)	3 (10)	
High	13 (43)	17 (57)		10 (33)	20 (67)		14 (47)	16 (53)		24 (80)	6 (20)	
k_ep_^4^			0.61			0.063			0.12			0.15
Low	16 (53)	14 (47)		15 (50)	15 (50)		17 (57)	13 (43)		28 (93)	2 (7)	
High	14 (47)	16 (53)		8 (27)	22 (73)		11 (37)	19 (63)		23 (77)	7 (23)	
v_e_^4^			0.3			0.43			0.038			1
Low	17 (57)	13 (43)		10 (33)	20 (67)		10 (33)	20 (67)		26 (87)	4 (13)	
High	13 (43)	17 (57)		13 (43)	17 (57)		18 (60)	12 (40)		25 (83)	5 (17)	
Blood flow^5^			0.42			0.008			0.054			0.32
Low	8 (62)	5 (38)		9 (69)	4 (31)		9 (69)	4 (31)		13 (100)	0 (0)	
High	19 (49)	20 (51)		10 (26)	29 (74)		15 (39)	24 (61)		33 (85)	6 (15)	
ADC^4^			0.18			0.032			0.038			0.51
High	14 (41)	20 (59)		18 (53)	16 (47)		21 (62)	13 (38)		27 (82)	6 (18)	
Low	19 (58)	14 (42)		9 (27)	24 (73)		12 (36)	21 (64)		30 (88)	4 (12)	

^1^Microvessel density/mm^2^, proliferating microvessel density/mm^2^ and vascular proliferation index (%) in pathological high-grade area, dichotomised by median.

^2^Glomeruloid microvascular proliferation.

^3^*P*-value, Pearson Chi-square or Fisher`s Exact Test.

^4^Median value of K^trans^ (min^-1^), median value of k_ep_ (min^-1^), median value of v_e_ and apparent diffusion coefficient (mm^2^/s) in pathological high-grade area, dichotomised by median.

^5^Mean value of blood flow (mL/100 g/min) in pathological high-grade area, dichotomised by lower quartile.

### Tissue-based microvascular proliferation and quantitative mpMRI-parameters are associated with adverse clinico-pathological features

High pMVD was borderline associated with high Gleason score (Gleason GG ≥ 4) and a cribriform Gleason pattern in the hot-spot area chosen for vessel counting (*p* = 0.063 and *p* = 0.050). Furthermore, high pMVD was significantly associated with extra-prostatic extension, high pathological stage, and large tumour dimension, as seen in Table 3. Presence of glomeruloid microvascular proliferation (GMP) was associated with most adverse clinico-pathological features (Table 3), as well as with high pMVD (*p* = 0.041). High pMVD, GMP, K^trans^, k_ep_, and blood flow were all associated with the D`Amico high-risk group.

**Table 3:** Associations between clinico-pathological variables, immunohistochemical markers of angiogenesis, and quantitative mpMRI-parameters.

Variables	pMVD^1^			GMP^2^			K^trans3^			k_ep_^3^			Blood flow^4^			ADC^5^		
	Low n (%)	High n (%)	*p*^6^	Absent n (%)	Present n (%)	*p*^6^	Low n (%)	High n (%)	*p*^6^	Low n (%)	High n (%)	*p*^6^	Low n (%)	High n (%)	*p*^6^	Low n (%)	High n (%)	*p*^6^
Gleason score^7^			0.43			0.060			0.024			0.005			0.044			0.16
≤ 3 + 4	20 (44)	26 (56)		42 (91)	4 (9)		25 (60)	17 (40)		26 (62)	16 (38)		12 (33)	24 (67)		20 (44)	26 (56)	
≥ 4 + 3	7 (33)	14 (67)		15(71)	6(29)		5 (28)	13 (72)		4 (22)	14 (78)		1 (6)	15 (94)		13 (62)	8 (38)	
Gleason score^8^			0.063			0.060			0.052			0.052			0.044			0.003
≤ 7	22 (48)	24 (52)		42 (91)	4 (9)		24 (59)	17 (41)		24 (59)	17 (41)		12 (33)	24 (67)		17 (37)	29 (63)	
≥ 8	5 (24)	16 (76)		15 (71)	6 (29)		6 (32)	13 (68)		6 (32)	13 (68)		1 (6)	15 (94)		16 (76)	5 (24)	
Cribriform pattern^8^			0.050			0.002			0.60			0.30			0.023			0.064
Absent	16 (53)	14 (47)		30 (100)	0 (0)		14 (54)	12 (46)		15 (58)	11 (42)		9 (41)	13 (59)		11 (37)	19 (63)	
Present	11 (30)	26 (70)		27 (73)	10 (27)		16 (47)	18 (53)		15 (44)	19 (56)		4 (13)	26 (87)		22 (60)	15 (40)	
Extra-prostatic extension			0.009			0.009			0.37			0.14			0.078			0.018
Absent	25 (49)	26 (51)		47(92)	4 (8)		24 (53)	21 (47)		25 (56)	20 (44)		12 (32)	25 (68)		21 (41)	30 (59)	
Present	2 (13)	14 (87)		10/63)	6 (37)		6 (40)	9 (60)		5 (33)	10 (67)		1 (7)	14 (93)		12 (75)	4 (25)	
Seminal vesicle invasion			1.00			0.22			1.00			1.00			1.00			0.11
Absent	25 (41)	36 (59)		53 (87)	8 (13)		28 (51)	27 (49)		28 (51)	27 (49)		12 (26)	35 (74)		28 (46)	33 (54)	
Present	2 (33)	4 (67)		4 (67)	2 (33)		2 (40)	3 (60)		2 (40)	3 (60)		1 (20)	4 (80)		5 (83)	1 (17)	
Pathological stage^9^			0.017			0.018			0.56			0.24			0.30			0.005
pT2	24 (49)	25 (51)		45 (92)	4 (8)		23 (52)	21 (48)		24 (55)	20 (45)		11 (31)	25 (69)		19 (39)	30 (61)	
≥ pT3	3 (17)	15 (83)		12 (67)	6 (33)		7 (44)	9 (56)		6 (38)	10 (62)		2 (13)	14 (87)		14 (78)	4 (22)	
Lymph node infiltration^10^			0.51			1.00			0.49			1.00			1.00			0.24
Absent^11^	27 (42)	38 (58)		55 (85)	10 (15)		28 (48)	30 (52)		29 (50)	29 (50)		13 (26)	37 (74)		31 (48)	34 (52)	
Present	0 (0)	2 (100)		2 (100)	0 (0)		2 (100)	0 (0)		1 (50)	1 (50)		0 (0)	2 (100)		2 (100)	0 (0)	
Tumour dimension^12^			0.028			0.013			0.54			0.54			0.47			0.14
Low	24 (48)	26 (52)		46(92)	4 (8)		24 (52)	22 (48)		24 (52)	22 (48)		11 (29)	27 (71)		22 (44)	28 (56)	
High	3 (18)	14(82)		11(65)	6 (35)		6 (43)	8 (57)		6 (43)	8 (57)		2 (14)	12 (86)		11 (65)	6 (35)	
Surgical margins			0.40			0.009			0.37			0.77			0.48			0.074
Negative	22 (43)	29 (57)		47 (92)	4 (8)		21 (47)	24 (53)		22 (49)	23 (51)		11 (28)	28 (72)		22 (43)	29 (57)	
Positive	5 (31)	11 (69)		10 (63)	6 (37)		9 (60)	6 (40)		8 (53)	7 (47)		2 (15)	11 (85)		11 (69)	5 (31)	
s-PSA^13^			0.22			1.00			0.045			0.15			1.00			0.73
Low	18 (36)	32 (64)		42 (84)	8 (16)		25 (58)	18 (42)		24 (56)	19 (44)		9 (24)	28 (76)		24 (48)	26 (52)	
High	9 (53)	8 (47)		15 (88)	2 (12)		5 (29)	12 (71)		6 (35)	11 (65)		4 (27)	11 (73)		9 (53)	8 (47)	
D`Amico			0.020			0.001			0.020			0.020			0.050			0.18
Low/Interm.risk	26 (47)	29 (53)		51 (93)	4 (7)		28 (57)	21 (43)		28 (57)	21(43)		13 (31)	29 (69)		25 (46)	30 (54)	
High-risk	1 (8)	11 (92)		6 (50)	6 (50)		2 (18)	9 (82)		2 (18)	9 (82)		0 (0)	10 (100)		8 (67)	4 (33)	

^1^Proliferating microvessel density/mm^2^ in pathological high-grade area, dichotomised by median.

^2^Glomeruloid microvascular proliferation.

^3^K^trans^ (min^-1^) and k_ep_ (min^-1^) in pathological high-grade area, dichotomised by median.

^4^Blood flow (mL/100 g/min) in pathological high-grade area, dichotomised by lower quartile.

^5^ADC (mm^2^/s) in pathological high-grade area, dichotomised by median.

^6^*p*-value, Pearson Chi-square or Fisher`s Exact Test.

^7^Gleason score in radical prostatectomy specimens.

^8^Gleason score/pattern in counted hot-spot area, pathological high-grade area.

^9^Pathological stage, UICC TNM Classification of malignant tumours, Eighth edition, 2017^59^.

^10^Pelvic lymph node infiltration at radical prostatectomy.

^11^Includes cases without lymphadenectomy.

^12^Largest tumour dimension in prostatectomy specimens, dichotomised by upper quartile (≥ 35 mm).

^13^Pre-operative s-PSA, dichotomised by upper quartile (s-PSA ≥ 15.8 ng/ml).

High K^trans^ and high k_ep_ and high blood flow were associated with high Gleason score (Gleason GG ≥ 3) in the surgical specimen (*p* ≤ 0.044), and high K^trans^ was also associated with high s-PSA (*p* = 0.045) (Table 3). Low ADC was associated with high Gleason score, extra-prostatic extension, and high pathological stage (Table 3). In contrast, MVD and v_e_ were not associated with any clinico-pathological factors. Using continuous variables (Mann–Whitney test), similar results were achieved (Supplementary Table S1).

### Tissue-based microvascular proliferation and quantitative mpMRI-parameters are associated with disease recurrence and metastasis

**Figure 1:**
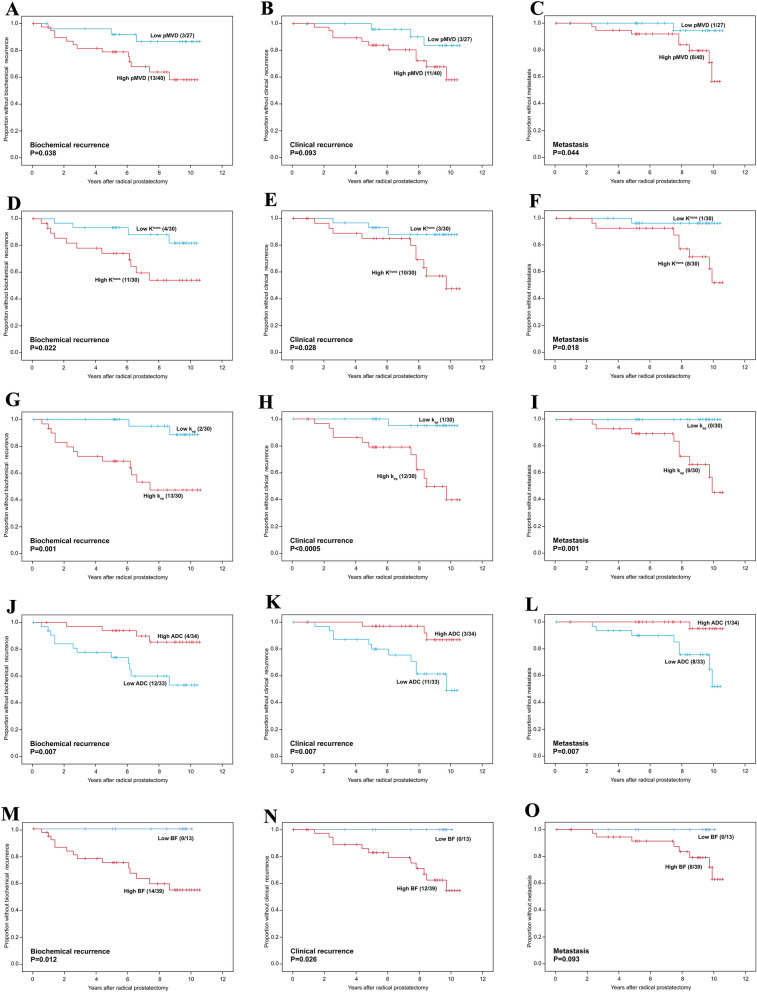
Univariate survival analyses (Kaplan–Meier) according to pMVD (**A**–**C**), K^trans^ (**D**–**F**), k_ep_ (**G**–**I**), ADC (**J**–**L**) and blood flow (**M**–**O**). End-points: Biochemical recurrence, clinical recurrence, and metastasis.

By univariate survival analysis, high pMVD, high K^trans^, high k_ep_, and low ADC were all significantly associated with shorter time to biochemical recurrence and metastasis. High K^trans^, high k_ep_, and low ADC were also significantly associated with clinical recurrence, whereas pMVD was borderline associated with this end-point. High tumour blood flow was associated with shorter time to biochemical and clinical recurrence (Supplementary Table S2, Fig. 1). Differences in survival were not found for MVD, presence of GMP, or v_e_. Comparable results were found using continuous variables in Cox’ univariate survival analyses (Supplementary Table S3).

Multivariate Cox’ survival analyses were performed using the end-points biochemical recurrence and clinical recurrence. When including the tissue-marker of angiogenesis from IHC (pMVD) together with quantitative parameters from mpMRI (K^trans^, k_ep_ and blood flow) (Table 4), pMVD independently predicted biochemical recurrence, with a borderline significance for clinical recurrence (HR 4.1 and 3.2, *p* = 0.032 and *p* = 0.093) together with k_ep_ (HR 7.9 and 13.4, *p* = 0.001 and *p* = 0.001).

**Table 4:** Multivariate survival analysis (Cox’ proportional hazards method) for pMVD, tumour blood flow, K^trans^, and k_ep_ in prostate cancer.

Variables	No	HR^1^	95% CI^2^	*P*-values^3^
Biochemical recurrence				
pMVD^4^				
Low	23	1.0		
High	37	4.1	0.9–18.4	0.032
k_ep_^4^				
Low	30	1		
High	30	7.9	1.8–35.8	0.001
Clinical recurrence				
pMVD^4^				
Low	23	1.0		
High	37	3.2	0.7–14.4	0.093
k_ep_^4^				
Low	30	1		
High	30	13.4	1.7–104.0	0.001

^1^Hazard ratio.

^2^Confidence interval.

^3^Likelihood ratio test.

^4^In pathological high-grade area, dichotomised by median.

The candidate markers from IHC and mpMRI were also included individually together with standard prognostic variables Gleason score (≥ GG3 [4 + 3] versus ≤ GG2 [3 + 4]), pathological stage (≥ pT3 vs pT2), and preoperative s-PSA (≥ 15.8 vs < 15.8, upper quartile) (Table 5). Univariate survival data for the standard variables are found in Supplementary Table S2. pMVD, K^trans^, k_ep_, and ADC independently predicted biochemical recurrence (HR 3.1–6.2, *p* = 0.007–0.069), whereas k_ep_ and ADC independently predicted clinical recurrence (HR 7.8 and 4.3, *p* = 0.016 and 0.014). Gleason score remained an independent predictor in all models (Table 5). Blood flow was not an independent predictor of any end-point.

**Table 5:** Multivariate survival analysis (Cox’ proportional hazards method) in prostate cancer.

pMVD^1^			K^trans1^			k_ep_^1^			ADC^1^		
Variables (n)	HR^2^ (95% CI^3^)	*P*-value^4^	Variables (n)	HR^2^(95% CI^3^)	*P*-value^4^	Variables (n)	HR^2^(95% CI^3^)	*P*-value^4^	Variables (n)	HR^2^(95% CI^3^)	*P*-value^4^
Biochemical recurrence			Biochemical recurrence			Biochemical recurrence			Biochemical recurrence		
Gleason score^5^			Gleason score^5^			Gleason score^5^			Gleason score^5^		
≤ 3 + 4 (46)	1.0		≤ 3 + 4 (42)	1.0		≤ 3 + 4 (42)	1.0		≤ 3 + 4 (46)	1.0	
≥ 4 + 3 (21)	16.7 (4.5–61.9)	< 0.0005	≥ 4 + 3 (18)	7.6 (1.9–30.0)	0.002	≥ 4 + 3 (18)	7.7 (2.0–28.8)	0.001	≥ 4 + 3 (21)	15.8 (4.3–58.7)	< 0.0005
Preop. s-PSA^6^			Path. Stage^7^			Path. Stage^7^			Preop. s-		
Low (50)	1.0		pT2 (44)	1.0		pT2 (44)	1.0		PSA^6^ Low (50)	1.0	
High (17)	4.8 (1.5–15.2)	0.008	≥ pT3 (16)	5.6 (1.7–18.8)	0.003	≥ pT3 (16)	5.6 (2.8–17.5)	0.003	High (17)	3.6 (1.2–10.7)	0.023
pMDV			K^trans^			k_ep_			ADC		
Low (27)	1.0		Low (30)	1.0		Low (30)	1.0		High (34)	1.0	
High (40)	5.4 (1.4–21.0)	0.007	High (30)	3.1 (0.8–11.2)	0.069	High (30)	6.2 (1.3–30.5)	0.009	Low (33)	4.4 (1.3–15.0)	0.009
						Clinical recurrence			Clinical recurrence		
						Gleason score^5^			Gleason score^5^		
						≤ 3 + 4 (42)	1.0		≤ 3 + 4 (46)	1.0	
						≥ 4 + 3 (18)	5.6 (1.5–21.4)	0.005	≥ 4 + 3 (21)	10.1 (2.8–36.3)	< 0.0005
						k_ep_			ADC		
						Low (30)	1.0		High (34)	1.0	
						High (30)	7.8 (1.0–64.6)	0.016	Low (33)	4.3 (1.2–15.7)	0.014

^1^In pathological high-grade area, dichotomised by median.

^2^Hazard ratio.

^3^Confidence interval.

^4^Likelihood ratio test.

^5^Gleason score in radical prostatectomy specimens.

^6^Preoperative s-PSA, cut off upper quartile (s-PSA ≥ 15.8 ng/ml).

^7^Pathological stage, UICC TNM Classification of malignant tumours, Eighth edition, 2017 ^59^.

### Combining tissue-based microvascular proliferation and quantitative mpMRI-parameters defines subgroups of patients with adverse tumour features and poor outcome

Subgroups were made by combining pMVD on IHC with quantitative mpMRI-parameters in matched anatomical areas. High pMVD combined with high k_ep_ was found in 22 patients, whereas high pMVD combined with high tumour blood flow was found in 29 patients. These subgroups were associated with high Gleason score, extra-prostatic extension, high pathological stage (pMVD + BF showing a trend, *p* = 0.063), presence of GMP, and the D`Amico high-risk group (Supplementary Table S4). The subgroups were strongly associated with shorter time to biochemical recurrence, clinical recurrence, and metastasis (*P* ≤ 0.036) by univariate survival analyses (Supplementary Fig. S1). In multivariate Cox’ survival analyses, both subgroups independently predicted biochemical recurrence (HR 5.5, *p* = 0.012; HR 8.8, *p* = 0.002) and clinical recurrence (HR 4.6, *p* = 0.017; HR 4.0, *p* = 0.047) together with Gleason score (Supplementary Table S5).

## Discussion

In this study, angiogenesis was assessed in prostate cancer by dual Nestin-Ki67 IHC along with the quantitative mpMRI-parameters K^trans^ and k_ep_ from anatomically matched tumour areas. In a subset of patients (n = 52), we also assessed tumour blood flow, by using a different model. We demonstrate that increased direct (static, by IHC) and indirect (dynamic, by mpMRI) measures of tumour-associated angiogenesis were associated with adverse clinico-pathological factors and disease progression, with a strong association between high microvascular proliferation and high tumour blood flow. In multivariate survival analyses, we found that microvascular proliferation was a stronger predictor compared with blood flow and that K_ep_ was a stronger predictor compared with K^trans^ and blood flow.

Studies investigating the relationship between MVD and basic estimates of angiogenesis from mpMRI have been previously performed on matched tumour areas in relatively small series of prostate cancer^29–32^. Some of the studies found a positive correlation between k_ep_ and MVD, others did not find any correlation^29–32^. However, microvascular proliferation is a better marker of active, ongoing angiogenesis and a better predictor of outcome than MVD^14,15,18–20^ and has to our knowledge not been investigated in prostate cancer together with imaging parameters.

In multivariate Cox’ survival analyses, pMVD, K^trans^, k_ep_, and ADC independently predicted biochemical recurrence, whereas k_ep_ and ADC independently predicted clinical recurrence, but blood flow was not an independent predictor of any end-point. However, a subgroup of patients with combined high pMVD and high tumor blood flow independently predicted both biochemical and clinical recurrence. Our results invite further studies to investigate the potential use of quantitative parameters from mpMRI as indirect in vivo markers of angiogenesis and tumour aggressiveness already at the time of diagnosis. These indirect markers of angiogenesis are more functional and might be combined with direct but static tissue-markers of angiogenesis from IHC for potential use in prognostication and risk stratification of prostate cancer patients.

Several studies have found a negative correlation between ADC and Gleason score in prostate cancer^29,34–38^, probably a reflection of increased tumour cellularity^29,37^, resulting in restricted movement of water molecules. Studies have also demonstrated an association between low ADC and reduced biochemical recurrence-free survival^39,40^. Furthermore, some studies demonstrate an inverse relationship between ADC and MVD^41,42^, VEGF^38,42^, or Hif-1α^38^ in prostate cancer, suggesting that ADC may be a potentially useful surrogate marker of angiogenesis as well. In concordance with this, low ADC was associated with high pMVD, aggressive tumour features, and reduced recurrence-free and metastasis-free survival in our material.

Our findings on pMVD validate the prognostic importance of microvascular proliferation in prostate cancer as found in a previous study presented by our group using an independent cohort^18^. The results are also in line with previous studies from our group on breast carcinoma^19^ and lung carcinoma^20^. However, direct assessment of angiogenesis is time-consuming with inter-observer variations. Further studies are needed to validate our findings and to establish a standardised and possibly automated method for pMVD-assessment for practical implementation.

GMPs are small blood vessels arranged in aggregates resembling renal glomeruli^43^. GMP is one of the diagnostic features of glioblastoma multiforme^44^, but have also been found as a prognostic marker in several other malignancies^45–48^. GMP is suggested to be a better prognostic marker than MVD^47,48^, and has been associated with lack of response to neoadjuvant chemotherapy in breast cancer^45^. In our study, presence of GMP was associated with increased pMVD and with most adverse clinico-pathological features, but not with outcome. Overall, pMVD was a better prognostic marker compared to GMP in our material.

This study has some limitations. It is a retrospective study with a relatively small sample size, hence results should be interpreted carefully. Assessment of MVD and pMVD is not standardised, and it is difficult to compare the results with other studies using different antibodies, different methods for vessel counting and different cut-off values. We decided to count vessels manually in hot-spot areas using established criteria^8–10,12,14–16,18–20^ originally described by Weidner et. al^5,6^. To obtain more objective measurements, counting in random areas or even counting in the whole tissue have been proposed^13^ as has the use of image analysis^11,13,29,30,32^. Another limitation of our study is the MRI procedure. The current standard at our hospital is to perform pre-biopsy mpMRI. In our study, mpMRI was performed preoperatively on patients with biopsy-proven prostate cancer and haemorrhage after the biopsy procedure may potentially have influenced the MRI parameters. However, with a median time delay of more than three months between biopsy and MRI, the risk for an influence from tissue trauma following biopsy is likely to be low. A strength of our study is the high anatomical correspondence between the ROIs for quantitative imaging parameters and the selected areas for IHC measurements. To reduce the problem of tumour heterogeneity, not being captured by a single ROI, the MRI parameters may also be analysed by pixel-by-pixel analysis of the whole tumour, requiring time consuming whole-volume tumour segmentations^28^. However, ROI seems to be better for model fitting^49^ and is frequently used^25–27,29–33^.

Two different softwares were used to extract the mpMRI data. Inter-software variation may be explained by different methods of estimating baseline T1^50^. We used the open-source software Quantiphyse to collect the basic quantitative parameters. Here, more patients were included, and this software has a robust method of estimating T1. NordicIce was primarily used to obtain data on tumour blood flow. However, extracted data correlated between softwares.

Although angiogenic activity is essential for cancer growth, progression, and metastasis, the use of anti-angiogenic agents for treatment has not been as straightforward as initially advocated^51,52^. Anti-angiogenic therapy has demonstrated some efficacy in clinical trials on hormone-sensitive prostate cancer patients^53–55^, but fails to improve overall survival for patients with castration resistance^54–56^. Our investigated markers of angiogenesis might aid in the prediction of response to anti-angiogenic treatment and could potentially be of value in patient stratification. The markers may also have the potential to aid in the evaluation of treatment effect and should be further studied in clinical trials of prostate cancer patients undergoing such therapy.

Angiogenesis is essential for tumour growth and metastasis. We here show that microvascular proliferation, by dual IHC with Nestin-Ki67, is superior to standard microvessel density to reflect active, ongoing angiogenesis and to predict patient outcome. DCE-MRI allows quantitative assessment of vascular permeability and perfusion and may hence characterize the dynamic tumour microvasculature and reflect tumour angiogenesis in vivo*,* as shown by standard quantitative parameters K^trans^ and k_ep_, as well as tumour blood flow. Of these mpMRI-acquired parameters, k_ep_ was the strongest predictor of outcome.

To summarise, we have demonstrated significant relations between microvascular proliferation by dual IHC with Nestin-Ki67 on radical prostatectomy specimens and in vivo quantitative parameters from preoperatively acquired mpMRI. These investigated markers of angiogenesis were associated with disease progression, microvascular proliferation being a stronger predictor compared with blood flow. The use of direct and indirect measures of angiogenesis, maybe in combination, has the potential to aid in the assessment of prognosis and treatment planning of prostate cancer patients.

## Materials and methods

### Patients and tissues

The study includes 67 prostate cancer patients, treated with robot-assisted laparoscopic radical prostatectomy (2010) at Haukeland University Hospital, Bergen, Norway. All patients underwent a pre-operative endorectal 1.5-T mpMRI. The DCE parameters K^trans^, k_ep_, and v_e_ were successfully quantified for 60/67 patients using the software Quantiphyse, whereas blood flow was successfully obtained for 52/67 patients, using NordicIce. All cases were acinar adenocarcinomas, including one case with partly pseudohyperplastic features and several cases with focal mucinous features. No patients received neo-adjuvant therapy except one patient who received bicalutamide a short period pre-operatively before deprescribing. Diagnostic biopsies were taken prior to mpMRI, the median number of biopsies was 10 (range 3–14) (Supplementary Table S6). Median time from biopsy to MRI was 99 days (mean 129 days, range 15–455). Median time from MRI to radical prostatectomy was 70 days (mean 67, range 1–224 days) and from biopsy to radical prostatectomy 173 days (mean 193, range 55–497). pMVD was not associated with time since biopsy (Pearson’s chi-square, *p* = 0.518) or number of biopsies taken (*p* = 0.228), and with one exception (Kep, *p* = 0.013), mpMRI parameters were not significantly associated with time since biopsy or number of biopsies taken. The entire prostate was routinely studied using whole-mount sections with 5 mm intervals, corresponding to the axial MR images. For each case, a radiologist (LARR) and two pathologists (KG and OJH) independently selected and registered the area of highest tumour grade by MRI and histopathology, respectively. Afterwards, the high-grade tumours were compared by looking at drawings from the whole-mount histological sections of the entire prostate and the at that time standard reporting scheme for locating 27 regions of interest on MRI^57,58^. The high-grade areas were then cut out of the original paraffin block, re-embedded in paraffin, and sectioned for IHC. In ten cases, the high-grade tumours were placed in different areas according to radiological and pathological examination. In these cases, both areas were cut out and examined without finding any significant differences between the areas regarding MVD, pMVD, VPI, K^trans^, k_ep_, v_e_, blood flow, or ADC (McNemar and Wilcoxon tests). These patients had only Gleason GG 1–3, ≤ cT2b tumours, but did otherwise not stand out from the others. Only results from the pathological high-grade area are used in this study. This study was approved by the Western Regional Committee for Medical and Health Research Ethics, REC West (REK 2015/2178) and the South Eastern Regional Committee for Medical and Health Research Ethics, REC South East (REK 2009/711). All patients gave their written informed consent. All methods were performed in accordance with guidelines and regulations by the University of Bergen and REK, and in accordance with the Declaration of Helsinki Principles.

### Clinico-pathological variables

Information regarding Gleason score, cribriform Gleason grade 4 pattern, extra-prostatic extension, seminal vesicle invasion, surgical margins, pelvic lymph node status at prostatectomy, and largest tumour dimension were recorded from the pathology records. The hot-spot area selected for vessel counting was also Gleason graded separately. Age at diagnosis, preoperative s-PSA, date of primary diagnosis, date of surgery, and clinical TNM stage^59^ were registered from the clinical patient files (Supplementary Table S6).

### Follow-up

The last date of follow-up was December 2020. The median follow-up was 102 months. Follow-up data on time from surgery until biochemical recurrence (s-PSA ≥ 0.2 ng/ml in two consecutive blood samples), clinical recurrence (development of any metastasis or locoregional recurrence), locoregional recurrence (a tumour in the prostatic fossa or a > 50% reduction of s-PSA or a s-PSA level < 0.1 ng/ml after local radiation therapy), metastases (identified on CT, MRI, or PSMA-PET CT), overall survival, and prostate cancer-specific survival were obtained from hospital records. At the last follow-up, 8 patients had died (two due to prostate cancer). There were 16 biochemical recurrences, 14 clinical recurrences, and 7 locoregional recurrences. 9 patients developed metastases (3 skeletal metastases, 2 of them with additional lymph node metastases, 5 lymph node metastases, and one soft tissue metastasis) (Supplementary Table S6). 11 patients received adjuvant radiation therapy due to positive surgical margins, extra-prostatic extension, lymph node metastases in the surgical specimens, or s-PSA persistence (6 patients). These patients were not counted as events from the beginning, but thoroughly reviewed regarding time to later biochemical recurrence, clinical recurrence, or metastasis. Survival analyses on biochemical recurrence were done on the whole group as well as on the subgroup excluding the patients with s-PSA persistence (n = 6) with mostly corresponding results (data not shown).

### Immunohistochemistry

Regular sections from formalin-fixed, paraffin-embedded tissue were deparaffinised with xylene, rehydrated in alcohol, and distilled water. Microwave antigen retrieval (20 min, 350 W) was obtained in target retrieval solution, pH 6.0 (Dako, Glostrup, Denmark). After cooling, the slides were transferred to an autostainer instrument (Dako). Dual endogenous enzyme blocking solution (Dako) was added for 7 min. The slides were incubated (60 min, room temperature) with both Nestin mouse IgG SC-23927 10c2 (Santa Cruz Biotechnology, Santa Cruz, CA, USA), diluted 1:50 and Ki67 rabbit IgG Clone SP6 (Thermo Scientific, Fremont, CA, USA), diluted 1:100. HRP anti-mouse EnVision (Dako) for Nestin and goat anti-rabbit IgG (H + L) alkaline phosphatase (Southern Biotech, Birmingham, AL, USA) for Ki67 in the ratio alkaline phosphatase:EnVision 1:100 was added for 30 min in room temperature. The last part of the staining procedure was done manually, using a humidifying chamber (Magnetic Immunostaining Tray, Cell Path, UK). The alkaline phosphatase was localised by Ferangie Blue™ Chromogen System (Biocare Medical, Concord, CA, USA) (19 min, room temperature) and HRP by AEC + Substrate Chromogen (Dako) (17 min, room temperature). Counterstaining was not done. Faramount aqueous mounting medium (Dako) was used to mount the slides. Sections from colon cancer tissue with known reactivity were used as positive controls. Negative controls were obtained by applying diluent without the primary antibody.

### Evaluation of the staining

**Figure 2:**
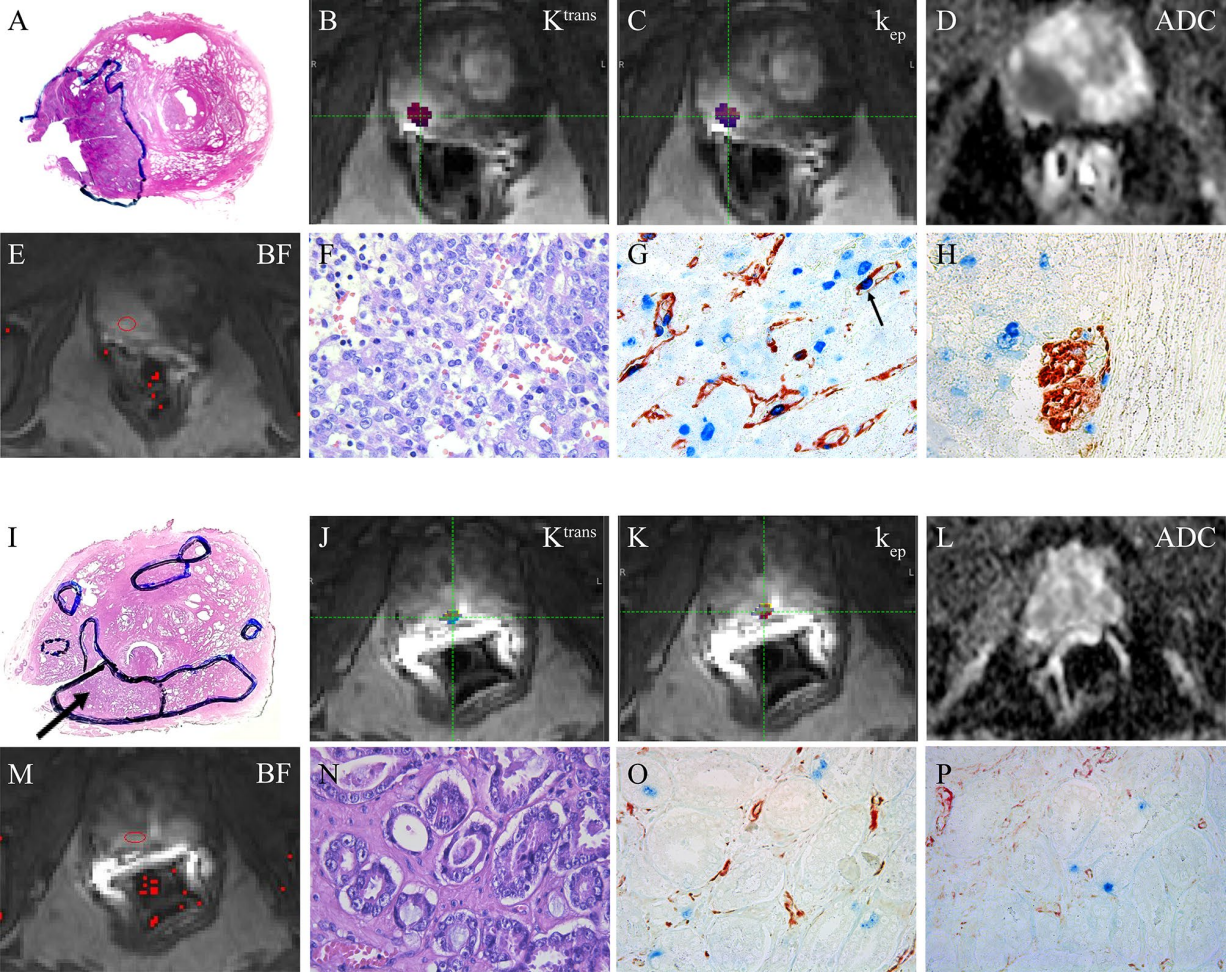
A-H: Patient (59 yr) treated with radical prostatectomy. Prostate cancer, pT3b, localised in the right peripheral zone outlined with an ink marker (**A**, photomicrograph of whole-mount HE-stained slide [lateral incision due to sampling of fresh tumour material]). In the selected ROI on DCE-MRI, median K^trans^ (**B**) was high (0.1434 min^-1^), median k_ep_ (**C**) was high (0.2088 min^-1^). Using NordicIce, tumour blood flow (**D**) in the selected ROI was high (12,06 mL/100 g/min). On DWI, ADC (**E**) was low (658 mm^2^/s). The tumour was graded as Gleason grade 4 + 5, score 9 (**F**, HE). Dual IHC staining by Nestin-Ki67 (**G**) showed high pMVD (33.72 proliferating vessels per mm^2^ (the arrow indicates a red Nestin-positive vessel with a blue Ki-67 positive endothelial nucleus) and presence of GMP (**H**). 6 years after treatment, the patient developed biochemical recurrence and was diagnosed with metastases to lymph nodes and soft tissue. (**I**–**O**) Patient (61 yr) treated with radical prostatectomy. Prostate cancer, pT2c, localised in the right peripheral zone outlined with an ink marker (**I**, photomicrograph of whole-mount HE-stained slide, arrow on the chosen lesion, [lateral incision due to sampling of fresh tumour material]). In the selected ROI on DCE-MRI, median K^trans^ (**J**) was low (0.0931 min^-1^), median k_ep_ (**K**) was low (0.1025 min^-1^). Using NordicIce, tumour blood flow (**L**) in the selected ROI was low (6.80 mL/100 g/min). On DWI, ADC (**M**) was high (1150 mm^2^/s). The tumour was graded as Gleason grade 3 + 3, score 6 (**N**, HE). Dual IHC staining by Nestin-Ki67 (**O**) showed 0 proliferating vessels per mm^2^ and no GMP (**P**). The patient had no biochemical or clinical recurrence during the follow-up. (Original magnification on the microscopic pictures: × 400).

The vessel counting method described by Weidner^5,6^ was used, blinded to MRI findings and patient information. The slides were evaluated at low magnification (× 40 and × 100) to find the most vascular areas (“hot-spots”) of the tumour. One hot-spot area per case was outlined with an ink marker. In hot-spot areas, 10 non-overlapping high-power fields (× 400, total area 2.46mm^2^) were examined for each case for the total number of Nestin-positive vessels. The number of stained vessels/mm^2^ was recorded as the MVD. The Nestin-positive structures needed to contain cells that resembled endothelial cells or clusters of endothelial cells to be counted. It should be clearly separate from adjacent microvessels. A vessel lumen was not required to be counted as a microvessel. Fibroblasts, nerves, and other non-endothelial cells in the stroma stained weaker than the endothelial cells and were not counted. The pMVD was evaluated in the same ten fields as MVD as the number of Nestin-positive vessels containing at least one proliferating endothelial cell/mm^2^. A proliferating endothelial cell was recognised by a blue Ki67-positive endothelial nucleus in a red Nestin-positive vessel (Fig. 2). Ki-67-positive nuclei within the vessel lumen or outside the vessel border were not included in the count. The vascular proliferation index (VPI) was calculated by dividing pMVD by MVD, as a percentage. GMP was defined as described earlier by Straume et al.^47^ and recorded as the presence or absence of glomerulus-like aggregates of multi-layered and closely associated Nestin-positive endothelial cells (Fig. 2). Presence of GMP was evaluated on the same histological slide as MVD and pMVD but was not necessarily found in the hot-spot area.

### Observer variability

Before evaluation of the slides in the cohort, a period of training was performed on a training set of colon carcinomas (n = 25) stained with CD31, Factor VIII, and Nestin-Ki67. As the dual staining had faded with time, only 11 cases could be used for Nestin-Ki67 counting. After a training period, Spearman`s rho for Nestin-Ki67 were 0.87 (*p* < 0.0005), 0.86 (*p* = 0.001), and 0.74 (*p* = 0.010) for MVD, pMVD, and VPI. Spearman`s rho for MVD by CD31 and Factor VIII were 0.86 (*p* < 0.0005) and 0.83 (*P* < 0.0005).

Intra-observer variability was tested in a blinded manner (AB) by evaluating the main series twice. For MVD, pMVD, and VPI, the Spearman`s rho and Kappa values (cut-off by median) were 0.89 (*p* < 0.0005), 0.82 (*p* < 0.0005), 0.83 (*p* < 0.0005), and 0.58, 0.81, and 0.70, respectively.

### MRI protocol

All images were acquired using a 1.5 T MR scanner (Avanto; Siemens Medical Systems, Erlangen, Germany) with an integrated endorectal phased-array coil (MR Innerva, Medrad, Pittsburgh, PA, USA). The scanning protocol has been published previously^58^. The mpMR-images (T2W, DWI, and DCE) covered the entire prostate gland with a temporal resolution of 6.16 s for the DCE.

### MRI interpretation and data analysis

Two observers (LARR, AR), with more than 3 years’ experience in reading prostate MRIs, read the MRI datasets. The matched areas for ROI placement within the predefined high-grade tumour areas were carefully selected by the radiologist (LARR) in cooperation with the pathologist who performed the IHC vessel assessment (AB). One tumour ROI was drawn per patient, except for the ten patients where two areas were assessed by IHC, here two ROIs were drawn. As a standard, three MRI planes through the tumour were included in the ROIs, one from the centre of the tumour focus and one from each side of the centre. All ROIs were segmented and analysed using Quantiphyse^60^, an Open Source software available from the University of Oxford. The quantitative parameters K^trans^, k_ep_, and v_e_ were obtained^61^ by applying the Tofts model^24^. The ADC values were collected from the high-grade tumours using DWI^58^. The ADC value was measured in the region of interest (ROI), including 2/3 of the lesion on axial ADC maps. Both mean, median, minimum, and maximum values of K^trans^, k_ep_, and v_e_ were recorded within each ROI. Median values were superior in the evaluation of all results, only median values are presented in this study.

Additionally, to obtain data on tumour blood flow, ROIs of all lesions were re-drawn by one radiologist (AR) and analysed using NordicIce v.4.1.3 (NordicNeuroLab Inc., Bergen, Norway). Mean tumour blood flow was calculated as the peak of the residue function. Furthermore, mean tumour k_ep_, K^trans^, and v_e_ were extracted (by the Tofts model), for comparison with the similar DCE parameters extracted in Quantiphyse^23^. The DCE parameters derived in NordicICE were all positively correlated to the comparable parameters derived in Quantiphyse (correlation coefficients ≥ 0.501 (*p* < 0.0005 for all), Supplementary Table S7), and yielded similar, but less significant results regarding associations with clinico-pathological features and patient outcome. For further analyses we hence incorporated the median tumour values of Ktrans, k_ep_, and v_e_ derived in Quantiphyse (n = 60), and the mean lesion blood flow derived in NordicIce (n = 52).

### Cut-off values

Median, tertile, and quartile values of the variables in the patient cohort were examined. The number of events and size of subgroups were considered and categories with comparable survival were merged. The median was selected as cut-off value for pMVD, K^trans^, k_ep_, and ADC. The lower quartile was used as cut-off for tumour blood flow. For pMVD, the median as cut-off value (0.00 vs ≥ 0.41) equalled absence/presence of pMVD and coincided with VPI using the lower quartile as cut-off. GMP was categorised as present or absent. s-PSA and the largest tumour dimension were dichotomised by the upper quartile.

### Statistics

The SPSS statistical package (IBM Corp., Armonk, NY, USA) version 27.0 was used for statistical analyses. The Spearman correlation test and Cohen’s kappa statistics were used to evaluate inter-and intra-observer agreement. The Spearman correlation test was used to analyse the relationship between continuous variables from quantitative mpMRI-parameters. Pearson’s chi-square or Fisher’s exact test were used to analyse associations between categorical variables. Associations between continuous and categorical variables were assessed by the Mann–Whitney *U* test. For related samples, the McNemar test and the Wilcoxon signed rank test were used. For univariate survival analyses, Kaplan–Meier plots were computed for visualisation and differences between the groups were analysed by the product-limit procedure (log-rank test). Cox’ univariate survival analyses were used for continuous variables. For multivariate survival analyses, the Cox’ proportional hazards method (likelihood ratio test for differences) was conducted, including variables with *p* < 0.10 in univariate analysis. Proportional hazard assumptions were examined by log–log plots.

### Supplementary Information

Supplementary Information.

## Data Availability

The data used for this study can be obtained from the corresponding author on reasonable request.
