# Supplementary material for: Microvascular proliferation is associated with high tumour blood flow by mpMRI and disease progression in primary prostate cancer
Source: Sci Rep. 2023 Oct 20;13:17949. doi: 10.1038/s41598-023-45158-4 (PMC10589248; doi:10.1038/s41598-023-45158-4)
Supplement: Supplementary file 1 — Supplementary Information. [file 41598_2023_45158_MOESM1_ESM.pdf]

Microvascular proliferation is associated with high tumour blood flow by mpMRI and disease progression in primary prostate cancer

*Astrid Børretzen[1,2], Lars A. R. Reisæter[3,4], Anders Ringheim[4,6], Karsten Gravdal[2], Svein A. Haukaas[5], Kristine E. Fasmer[3,6], Ingfrid H. S. Haldorsen[3,6], Christian Beisland[3,5], Lars A. Akslen[1,2], Ole J. Halvorsen[1]

[1]Centre for Cancer Biomarkers CCBIO, Gade Laboratory for Pathology, Department of Clinical Medicine, University of Bergen, Norway.

[2]Department of Pathology, Haukeland University Hospital, Bergen, Norway.

[3]Department of Clinical Medicine, University of Bergen, Norway.

[4]Department of Radiology, Haukeland University Hospital, Bergen, Norway.

[5]Department of Urology, Haukeland University Hospital, Bergen, Norway.

[6]Mohn Medical Imaging and Visualization Centre (MMIV), Department of Radiology, Haukeland University Hospital, Bergen, Norway

Supplementary Material

Supplementary Table S1 Associations between clinico-pathological variables, immunohistochemical markers of angiogenesis, and quantitative mpMRI-parameters (as continuous variables)

Supplementary Table S2 Survival analyses for standard prognostic variables, immunohistochemical markers of angiogenesis, and quantitative mpMRI-parameters

Supplementary Table S3 Univariate survival analyses (Cox' proportional hazards method) of immunohistochemical markers of angiogenesis and quantitative mpMRI-parameters (as continuous variables)

Supplementary Table S4 Associations between clinico-pathological variables and the combination of pMVD and k_{ep} or pMVD and tumour blood flow

Supplementary Table S5 Multivariate survival analysis (Cox' proportional hazards method) according to the combination of pMVD and k_{ep} or mMVD and tumour blood flow in prostate cancer

Supplementary Table S6 Clinico-pathological characteristics and follow-up status in patients with prostatic carcinomas (67 radical prostatectomies)

Supplementary Table S7 Correlations between quantitative mpMRI-parameters collected from Quantiphyse and NordicIce

Supplementary Figure S1 Univariate survival analyses (Kaplan-Meier) according to the combination of pMVD and k_{ep} (A-D) and the combination of pMVD and tumour blood flow (E-H). End-points: Biochemical recurrence, clinical recurrence, locoregional recurrence and metastasis

Supplementary Table S1 Associations between clinico-pathological variables, immunohistochemical markers of angiogenesis, and quantitative mpMRI-parameters (as continuous variables)

	pMVD[1]			K^{trans}[1]			k_{ep}[1]			ADC[1]			BF[1]		
Variables	Mean	Median	P-value[2]	Mean	Median	P-value[2]	Mean	Median	P-value[2]	Mean	Median	P-value[2]	Mean	Median	P-value[2]
Gleason score[3]			0.21			0.057			0.024			0.054			0.041
≤3+4	0.55	0.41		0.16	0.12		0.25	0.14		848.70	827.00		8.92	7.99	
≥4+3	2.75	0.41		0.29	0.16		0.40	0.20		717.05	721.00		11.30	10.33	
Gleason score[3]			0.027			0.15			0.023			0.012			0.024
≤7	0.59	0.41		0.18	0.13		0.27	0.16		831.40	827.00		9.02	8.52	
≥8	6.79	1.22		0.40	0.22		0.55	0.27		602.00	621.00		15.55	12.06	
Gleason score[4]			0.064			0.040			0.12			0.048			0.003
≤3+4	0.42	0.00		0.18	0.09		0.31	0.12		869.85	837.00		8.01	6.86	
≥4+3	1.79	0.41		0.21	0.15		0.28	0.19		765.30	778.00		10.67	9.91	
Gleason score[4]			0.14			0.091			0.026			0.001			0.045
≤7	0.60	0.41		0.16	0.11		0.25	0.15		865.54	839.50		8.91	8.36	
≥8	2.63	0.41		0.28	0.15		0.39	0.20		680.14	706.00		11.31	10.35	
Cribriform Gleason pattern[4]			0.016			0.65			0.45			0.034			0.35
Absent	0.38	0.00		0.20	0.11		0.31	0.15		886.87	832.50		9.98	7.71	
Present	1.93	0.41		0.20	0.14		0.28	0.17		743.03	776.00		9.41	8.85	
Extra-prostatic extension			0.013			0.54			0.17			0.004			0.031
Absent	0.54	0.41		0.20	0.13		0.29	0.16		851.86	833.00		9.28	8.27	
Present	3.45	0.61		0.21	0.15		0.31	0.19		665.91	660.50		10.56	10.33	
Seminal vesicle invasion			0.42			0.87			0.70			0.18			0.085
Absent	1.15	0.41		0.21	0.13		0.30	0.17		818.33	826.00		9.36	8.52	
Present	2.17	0.81		0.15	0.17		0.19	0.20		696.67	750.50		12.40	13.61	
Pathological stage[5]			0.017			0.78			0.26			0.004			0.078
pT2	0.54	0.41		0.20	0.13		0.29	0.16		855.61	834.00		9.38	8.36	
≥pT3	3.14	0.61		0.20	0.15		0.29	0.19		676.28	672.00		10.25	10.32	
Lymph node infiltration[6]			0.094			0.46			0.93			0.17			0.70
Absent[7]	1.22	0.41		0.20	0.13		0.30	0.17		812.52	817.00		9.65	8.58	
Present	1.83	1.83		0.10	0.10		0.17	0.17		642.00	642.00		9.51	9.51	
Tumour dimension[8]			0.077			0.69			0.86			0.080			0.052
Low	0.57	0.41		0.23	0.13		0.33	0.17		839.72	830.50		9.34	8.36	
High	3.20	0.41		0.12	0.14		0.18	0.19		712.47	780.00		10.48	10.35	
Surgical margins			0.24			0.25			0.52			0.20			0.70
Negative	0.56	0.41		0.23	0.13		0.34	0.17		826.84	828.00		9.74	8.58	
Positive	3.40	0.41		0.12	0.12		0.17	0.13		745.56	709.00		9.37	8.44	
PSA[9]			0.45			0.041			0.11			0.59			0.071
Low	1.30	0.41		0.19	0.11		0.29	0.15		803.62	821.50		8.82	8.44	
High	1.05	0.00		0.23	0.17		0.31	0.19		818.65	799.00		11.69	10.37	
D`Amico			0.002			0.004			0.010			0.034			0.004
Low/Intermediate	0.49	0.41		0.16	0.11		0.24	0.15		836.09	826.00		8.75	8.36	
High	4.64	1.02		0.41	0.20		0.55	0.21		676.08	647.50		13.43	11.53	

[1]In pathological high-grade area, continuous variables
[2]Mann-Whitney test
[3]Gleason score in radical prostatectomy specimens
[4]Gleason score/pattern in counted hot-spot area, pathological high-grade area
[5]Pathological stage, UICC TNM Classification of malignant tumours, Eighth edition, 2017
[6]Pelvic lymph node infiltration at radical prostatectomy
[7]Includes cases without lymphadenectomy
[8]Largest tumour dimension in prostatectomy specimens, dichotomised by upper quartile (≥35 mm)
[9]Pre-operative s-PSA, dichotomised by upper quartile (s-PSA ≥15.8 ng/ml)

Supplementary Table S2 Survival analyses for standard prognostic variables, immunohistochemical markers of angiogenesis, and quantitative mpMRI-parameters

Variables	No of patients	No of events	Est. 5 yrs. survival (%)	Est. 10 yrs. survival (%)	P-value[1]
**Biochemical recurrence**					
Gleason score[2]					<0.0005
≤3+4	46	3	95.6	92.5	
≥4+3	21	13	57.3	19.8	
Pathological stage[3]					<0.0005
pT2	49	6	93.7	84.7	
≥pT3	18	10	55.8	24.8	
Preop. s-PSA[4]					0.051
Low	50	9	91.3	74.1	
High	17	7	64.7	58.2	
pMVD[5]					0.038
Low	27	3	92.0	86.6	
High	40	13	78.9	58.2	
K^{trans}[5]					0.022
Low	30	4	93.3	81.9	
High	30	11	74.2	54.2	
k_{ep}[5]					0.001
Low	30	2	100.0	88.7	
High	30	13	69.0	47.4	
Blood flow[6]					0.012
Low	13	0	100.0	100.0	
High	39	14	75.0	54.6	
ADC[5]					0.007
High	34	4	93.9	85.4	
Low	33	12	73.9	53.4	
**Clinical recurrence**					
Gleason score[2]					<0.0005
≤3+4	46	3	97.8	91.3	
≥4+3	21	11	67.4	25.0	
Pathological stage[3]					<0.0005
pT2	49	5	95.7	85.7	
≥pT3	18	9	67.7	21.2	
Preop. s-PSA[4]					0.039
Low	50	7	91.2	80.5	
High	17	7	82.4	45.4	
pMVD[5]					0.093
Low	27	3	95.8	83.8	
High	40	11	84.1	58.3	
K^{trans}[5]					0.028
Low	30	3	93.1	87.9	
High	30	10	85.0	47.5	
k_{ep}[5]					<0.0005
Low	30	1	100.0	95.0	
High	30	12	79.0	39.7	
Blood flow[6]					0.026
Low	13	0	100.0	100.0	
High	39	12	83.0	54.8	
ADC[5]					0.007
High	34	3	97.0	86.8	
Low	33	11	79.8	49.0	
**Locoregional recurrence**					
Gleason score[2]					0.010
≤3+4	46	2	97.8	94.7	
≥4+3	21	5	78.3	69.6	
Pathological stage[3]					0.030
pT2	49	3	95.7	92.2	
≥pT3	18	4	80.8	72.7	
Preop. s-PSA[4]					0.31
Low	50	4	93.4	89.7	
High	17	3	88.2	81.9	
pMVD[5]					0.47
Low	27	2	95.8	89.4	
High	40	5	89.4	86.0	
K^{trans}[5]					0.36
Low	30	2	96.7	91.6	
High	30	4	88.7	82.8	
k_{ep}[5]					0.087
Low	30	1	100.0	95.0	
High	30	5	86.1	79.4	

Blood flow[6]					
Low	13	0	100.0	100.0	
High	39	6	88.7	80.5	
ADC[5]					0.17
High	34	2	97.0	91.9	
Low	33	5	86.7	82.4	
**Metastases**					
Gleason score[2]					<0.0005
≤3+4	46	1	100.0	96.6	
≥4+3	21	8	83.9	37.3	
Pathological stage[3]					<0.0005
pT2	49	2	100.0	93.4	
≥pT3	18	7	80.8	23.9	
Preop. s-PSA[4]					0.016
Low	50	3	97.7	90.7	
High	17	6	88.2	48.9	
pMVD[5]					0.044
Low	27	1	100.0	94.4	
High	40	8	92.0	56.6	
K^{trans}[5]					0.018
Low	30	1	96.3	96.3	
High	30	8	92.6	51.9	
k_{ep}[5]					0.001
Low	30	0	100.0	100.0	
High	30	9	89.4	45.6	
Blood flow[6]					0.093
Low	13	0	100.0	100.0	
High	39	8	91.4	62.9	
ADC[5]					0.007
High	34	1	100.0	95.0	
Low	33	8	89.8	51.9	

[1]Log-rank test
[2]Gleason score in radical prostatectomy specimens
[3]Pathological stage, UICC TNM Classification of malignant tumors, Eighth edition, 2017
[4]Preoperative s-PSA, dichotomised by upper quartile (s-PSA≥15.8)
[5]In pathological high-grade area, dichotomised by median
[6]In pathological high-grade area, dichotomised by lower quartile

Supplementary Table S3 Univariate survival analyses (Cox' proportional hazards method) of immunohistochemical markers of angiogenesis and quantitative mpMRI-parameters (as continuous variables)

Variables	No	HR[1]	95 % CI[2]	P-value[3]
**Biochemical recurrence**				
pMVD[4]	67	1.059	1.004-1.118	0.037
K^{trans}[4]	60	3.937	1.218-12.724	0.022
k_{ep}[4]	60	2.068	0.949-4.507	0.067
ADC[4]	67	0.995	0.992-0.998	0.001
BF[4]	52	1.068	0.997-1.144	0.060
**Clinical recurrence**				
pMVD[4]	67	1.058	1.001-1.118	0.044
K^{trans}[4]	60	5.350	1.528-18.735	0.009
k_{ep}[4]	60	2.657	1.170-6.032	0.020
ADC[4]	67	0.995	0.992-0.998	0.002
BF[4]	52	1.043	0.952-1.143	0.37
**Locoregional recurrence**				
pMVD[4]	67	1.021	0.908-1.147	0.73
K^{trans}[4]	60	4.301	0.781-23.685	0.094
k_{ep}[4]	60	2.212	0.710-6.886	0.17
ADC[4]	67	0.997	0.993-1.001	0.13
BF[4]	52	0.994	0.850-1.163	0.94
**Metastases**				
pMVD[4]	67	1.083	1.022-1.148	0.007
K^{trans}[4]	60	4.055	1.103-14.911	0.035
k_{ep}[4]	60	2.345	1.014-5.424	0.046
ADC[4]	67	0.993	0.989-0.997	0.001
BF[4]	52	1.090	0.986-1.205	0.094

[1]Hazard ratio
[2]Confidence interval
[3]Likelihood ratio test
[4]In pathological high-grade area

Supplementary Table S4 Associations between clinico-pathological variables and the combination of pMVD and k_{ep} or pMVD and tumour blood flow

Variables	pMVD and k_{ep}[1] Others n (%)	Both high n (%)	P-value[3]	pMVD and BF[2] Others n (%)	Both high n (%)	P-value[3]
Gleason score[4]			0.002			0.21
≤3+4	32 (76)	10 (24)		18 (50)	18 (50)	
≥4+3	6 (33)	12 (67)		5 (31)	11 (69)	
Gleason score[5]			0.004			0.002
≤7	31 (76)	10 (24)		21 (58)	15 (42)	
≥8	7 (37)	12 (63)		2 (13)	14 (87)	
Cribriform Gleason pattern[5]			0.17			0.016
Absent	19 (73)	7 (27)		14 (64)	8 (36)	
Present	19 (56)	15 (44)		9 (30)	21 (70)	
Extra-prostatic extension			0.005			0.025
Absent	33 (73)	12 (27)		20 (54)	17 (46)	
Present	5 (33)	10 (67)		3 (20)	12 (80)	
Seminal vesicle invasion			0.35			1.00
Absent	36 (66)	19 (34)		21 (45)	26 (55)	
Present	2 (40)	3 (60)		2 (40)	3 (60)	
Pathological stage[6]			0.012			0.063
pT2	32 (73)	12 (27)		19 (53)	17 (47)	
≥pT3	6 (38)	10 (62)		4 (25)	12 (75)	
Lymph node infiltration[7]			1.00			0.50
Absent[8]	37 (64)	21 (36)		23 (46)	27 (54)	
Present	1 (50)	1 (50)		0 (0)	2 (100)	
Tumour dimension[9]			0.069			0.044
Low	32 (70)	14 (30)		20 (53)	18 (47)	
High	6 (43)	8 (57)		3 (21)	11 (79)	
Surgical margins			0.76			0.63
Negative	29 (64)	16 (36)		18 (46)	21 (54)	
Positive	9 (60)	6 (40)		5 (39)	8 (61)	
s-PSA[10]			0.46			0.40
Low	26 (61)	17 (39)		15 (41)	22 (59)	
High	12 (71)	5 (29)		8 (53)	7 (47)	
D`Amico			0.012			0.029
Low/Intermediate	35 (71)	14 (29)		21 (53)	19 (47)	
High	3 (27)	8 (73)		1 (10)	9 (90)	
GMP			0.009			0.028
Absent	36 (71)	15 (29)		23 (50)	23 (50)	
Present	2 (22)	7 (78)		0 (0)	6 (100)	

[1]Proliferating microvessel density/mm^2 and k_{ep} (min^{-1}) or blood flow in pathological high-grade area, dichotomised by median
[2]Proliferating microvessel density/mm^2 and tumour blood flow (mL/100g/min) in pathological high-grade area, dichotomised by median (pMVD) or lower quartile (BF)
[3]Pearson Chi-square or Fisher`s Exact Test
[4]Gleason score in radical prostatectomy specimens
[5]Gleason score/pattern in counted hot-spot area, pathological high-grade area
[6]Pathological stage, UICC TNM Classification of malignant tumours, Eighth edition, 2017
[7]Pelvic lymph node infiltration at radical prostatectomy
[8]Includes cases without lymphadenectomy
[9]Largest tumour dimension in prostatectomy specimens, dichotomised by upper quartile (≥35 mm)
[10]Pre-operative s-PSA, dichotomised by upper quartile (s-PSA ≥15.8 ng/ml)

Supplementary Table S5 Multivariate survival analysis (Cox' proportional hazards method) according to the combination of pMVD and k_{ep} or mMVD and tumour blood flow in prostate cancer

pMVD and k_{ep}[1]			pMVD and BF[2]		
Variables (n)	HR[3] (95 % CI[4])	P-value[5]	Variables (n)	HR[3] (95 % CI[4])	P-value[5]
**Biochemical recurrence**			**Biochemical recurrence**		
Gleason score[6]			Gleason score[6]		
≤3+4 (42)	1.0		≤3+4 (36)	1.0	
≥4+3 (18)	8.5 (2.2-33.5)	0.001	≥4+3 (16)	11.6 (3.1-43.8)	<0.0005
Preop. s-PSA[7]			Preop. s-PSA[7]		
Low (43)	1.0		Low (37)	1.0	
High (17)	3.1 (0.9-10.5)	0.067	High (15)	4.5 (1.3-15.1)	0.016
Path. Stage[8]					
pT2 (44)	1.0				
≥pT3 (16)	3.0 (0.9-10.2)	0.080			
pMDV and k_{ep}			pMDV and BF		
Others (38)	1.0		Others (23)	1.0	
Both high (22)	5.5 (1.3-23.0)	0.012	Both high (29)	8.8 (1.7-44.6)	0.002
**Clinical recurrence**			**Clinical recurrence**		
Gleason score[6]			Gleason score[6]		
≤3+4 (42)	1.0		≤3+4 (36)	1.0	
≥4+3 (18)	6.2 (1.6-24.0)	0.003	≥4+3 (16)	7.6 (2.0-28.3)	0.001
pMDV and k_{ep}			pMDV and BF		
Others (38)	1.0		Others (23)	1.0	
Both high (22)	4.6 (1.2-18.0)	0.017	Both high (29)	4.0 (0.9-18.4)	0.047

[1]Proliferating microvessel density/mm^2 and k_{ep} (min^{-1}) or blood blow (BF) in pathological high-grade area, dichotomised by median
[2]Proliferating microvessel density/mm^2 and tumour blood flow (mL/100g/min) in pathological high-grade area, dichotomised by median (pMVD) or lower quartile (BF)
[3]Hazard ratio
[4]Confidence interval
[5]Likelihood ratio test
[6]Gleason score in radical prostatectomy specimens
[7]Preoperative s-PSA, cut off by the upper quartile (s-PSA ≥15.8 ng/ml)
[8]Pathological stage, UICC TNM Classification of malignant tumours, Eighth edition, 2017

Supplementary Table S6 Clinico-pathological characteristics and follow-up status in patients with prostatic carcinomas (67 radical prostatectomies)

No. of patients	67
Age	
Mean	61
Median	62
Min-max	43-70
Preoperative s-PSA	
Mean	12.2
Median	9.5
Min-Max	1.6-54.9
Number of pre-operative biopsies	
Mean	9.8
Median	10.0
Min-Max	3-14
Gleason score[1] (%)	
3+3	19 (28)
3+4	27 (40)
4+3	14 (21)
3+5	1 (2)
4+4	2 (3)
4+5	4 (6)
Cribriform Gleason grade 4 (%)	
Absent	21 (31)
Present	46 (69)
Extra-prostatic extension (%)	
Absent	51 (76)
Present	16 (24)
Seminal vesicle invasion (%)	
Absent	61 (91)
Present	6 (9)
Pathological stage[2] (%)	
pT2	49 (73)
≥pT3	18 (27)
Clinical stage[3] (%)	
cT1c	40 (60)
cT2a	10 (15)
cT2b	11 (16)
cT2c	2 (3)
cT3a	4 (6)
Lymph nodes positive (%) of cases with lymph nodes removed (n=31)	2 (6)
Tumour dimension[4]	
Mean	26.2
Median	25.0
Min-Max	5-40
Surgical margins (%)	
Negative	51 (76)
Positive	16 (24)
Biochemical recurrence (%)	16 (24)
Clinical recurrence (%)	14 (21)
Locoregional recurrence (%)	7 (10)
Metastases (%)	9 (13)
Cancer-specific death (%)	2 (3)
All deaths (%)	8 (12)

[1]Gleason score in radical prostatectomy specimens
[2]Pathological stage, UICC TNM Classification of malignant tumours, Eighth edition, 2017
[3]Clinical stage, UICC TNM Classification of malignant tumours, Eighth edition, 2017
[4]Largest tumour dimension (mm) in prostatectomy specimens

Supplementary Table S7 Correlations between quantitative mpMRI-parameters collected from Quantiphyse and NordicIce

	K^{trans} [1]		k_{ep} [1]		v_e [1]	
Variables	Correlation coefficient[2]	p-value	Correlation coefficient[2]	p-value	Correlation coefficient[2]	p-value
K^{trans} [3]	0.577	<0.0005				
k_{ep} [3]			0.501	<0.0005		
v_e [3]					0.589	<0.0005

[1]Median value of K^{trans} (min^{-1}), median value of k_{ep} (min^{-1}), and median value of v_e in pathological high-grade area, collected from Quantiphyse
[2]Spearman's rank correlation coefficient (rho)
[3]Mean value of K^{trans} (min^{-1}), mean value of k_{ep} (min^{-1}), and mean value of v_e in pathological high-grade area, collected from NordicIce

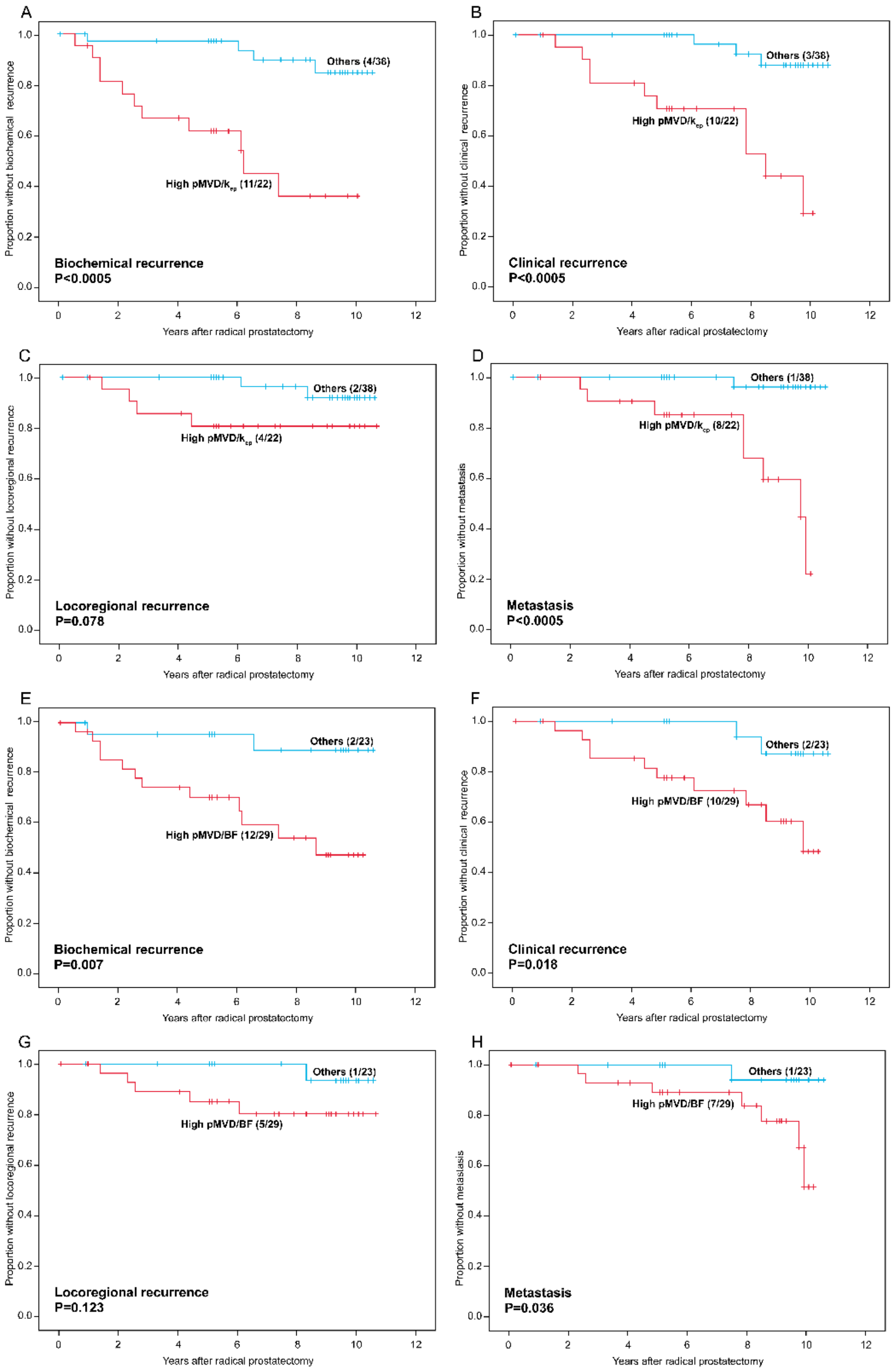

Supplementary Figure S1
